# Genetic resources and breeding approaches for improvement of amaranth (*Amaranthus* spp.) and quinoa (*Chenopodium quinoa*)

**DOI:** 10.3389/fnut.2023.1129723

**Published:** 2023-07-24

**Authors:** Anuradha, Manisha Kumari, Gaurav Zinta, Ramesh Chauhan, Ashok Kumar, Sanatsujat Singh, Satbeer Singh

**Affiliations:** ^1^Division of Agrotechnology, Council of Scientific and Industrial Research–Institute of Himalayan Bioresource Technology, Palampur, Himachal Pradesh, India; ^2^Division of Biotechnology, Council of Scientific and Industrial Research–Institute of Himalayan Bioresource Technology, Palampur, Himachal Pradesh, India

**Keywords:** genetic improvement, grain amaranth, quinoa, germplasm, nutrition

## Abstract

Nowadays, the human population is more concerned about their diet and very specific in choosing their food sources to ensure a healthy lifestyle and avoid diseases. So people are shifting to more smart nutritious food choices other than regular cereals and staple foods they have been eating for a long time. Pseudocereals, especially, amaranth and quinoa, are important alternatives to traditional cereals due to comparatively higher nutrition, essential minerals, amino acids, and zero gluten. Both Amaranchaceae crops are low-input demanding and hardy plants tolerant to stress, drought, and salinity conditions. Thus, these crops may benefit developing countries that follow subsistence agriculture and have limited farming resources. However, these are underutilized orphan crops, and the efforts to improve them by reducing their saponin content remain ignored for a long time. Furthermore, these crops have very rich variability, but the progress of their genetic gain for getting high-yielding genotypes is slow. Realizing problems in traditional cereals and opting for crop diversification to tackle climate change, research should be focused on the genetic improvement for low saponin, nutritionally rich, tolerant to biotic and abiotic stresses, location-specific photoperiod, and high yielding varietal development of amaranth and quinoa to expand their commercial cultivation. The latest technologies that can accelerate the breeding to improve yield and quality in these crops are much behind and slower than the already established major crops of the world. We could learn from past mistakes and utilize the latest trends such as CRISPR/Cas, TILLING, and RNA interference (RNAi) technology to improve these pseudocereals genetically. Hence, the study reviewed important nutrition quality traits, morphological descriptors, their breeding behavior, available genetic resources, and breeding approaches for these crops to shed light on future breeding strategies to develop superior genotypes.

## 1. Introduction

Crops that store carbohydrates in the perisperm are called Pseudocereals, unlike true cereals, where carbohydrates are stored in the endosperm and both are sources of energy in the human diet (1). These days with the health-conscious human population, people are more interested in healthier diet opportunities and more focused on micronutrient supply to their food other than usual food sources through major cereal crops. With the increasing trend to add more nutrition to the diet, the rise in demand for pseudocereals, grain amaranth, and quinoa's popularity is increasing daily in the market, resulting in their increasing cultivation (2, 3). Therefore, to meet the demand, there is a need for genetic improvement of these crops. Amaranth and quinoa are gaining space in the markets among other grains such as Buckwheat, Chia, and Wattle seeds due to their high nutritional qualities (4).

The grain amaranth (*Amaranthus* spp.) is valued as leaf vegetables, nutritional grain, and ornamentals by people worldwide. The “Amaranth” is a Greek word called “everlasting” as it is hardy; thus, it is famous for cultivation. It is the main staple food in many parts of the world such as Mexico, and it is consumed as a multipurpose crop in the form of grain and leaves, and in many places as forage for livestock using some amaranths (5). It is also used for natural dyes, as lubricants, and in the pharmaceutical industry. Amaranth flour is gluten-free, so it is a good food source for people with gluten allergies. Amaranth leaves are anti-cancerous, it stops the irregular growth of cancer cells in the breast, colon, and liver, thus are also suitable for cancer patients (6). In addition, many products are famous in the market made from amaranth grains (7). Among dicotyledonous plants, amaranth is one example that uses the C4 pathway for photosynthesis and could be a model plant in genetic regulation studies for photosynthesis (8). Amaranth is resistant to all abiotic and biotic stresses, including stress, drought, and insect-pest attacks, and needs less care, thus easy to cultivate in a wide range of agro-climatic regions (9). It is thought of as a saprophytic halophyte and is tolerant to high salinity; thus, it is suited for subsistence agriculture (9).

Another one, quinoa (*Chenopodium quinoa* Willd.) is a nutritious grain modern crop and old enough to have originated from the Andean area of South America (10). It was cultivated in earlier times in countries such as the Andean region, Bolivia, Peru, and Chile. About 7,000 years ago, the report of the first domestication was from Lake Titicaca, from where it spread further from South America and to around the world (11, 12). In Chile, cultivation of the quinoa plant began as early as 5,000 BC. Presently, about 250 different *Chenopodium* species are known all over the world (13). Since then, it has been a major staple grain for the Inca civilization, known in their native Quechua language as “chisiya mama” or “Mother Grain.” It is categorized either on the plant's color, shape, and fruit (14, 15). In the Andean region, quinoa has various names within the same community; it was famous for names such as kinua, quinhua, and jupha in the Aymara language, depending on the diversity in the color of its grains. Quinoa grows well in poor soil of rainfed conditions, has superior adaptability to varied agro-climatic conditions, and has unrealized commercial potential in India (16, 17). Quinoa's exceptional adaptability to various agro-ecological zones can be grown in all regions (2, 18) with water scarcity, from hot to dry deserts and in arid, semi-arid, and even tropical locations with humidity up to 88% and temperatures ranging from 8 to 40°C (19). It can also grow on varied topography, both in plains or high mountainous regions up to sea level (4,000 m), and thus it is a significant crop from an agricultural point of view to grow in a wide range of regions (18). Numerous European nations took part in the 1993-approved initiative titled “Quinoa-A Multipurpose Crop” which was most popular to be used for “Agricultural Diversification” (20). Quinoa is super famous these days in modern supermarkets due to its super nutritional quality of grains and leaves and is available as processed products in fancy packaging to attract customers as an alternate food to regular sources of carbohydrates instead of major cereals in the modern diet.

Food security is a major concern for future generations. These crops can be grown in most countries' environmental conditions and are easily available plant sources of micronutrients to alleviate micronutrient malnutrition (21). There is a need for their genetic improvement and to explore the breeding behavior of these crops, possibilities to create variations in population, and the use of molecular markers to study diversity in amaranth and quinoa (22–24) (Table 3). We have reviewed genetic mapping studies and DNA barcoding to understand the gene sequence of these crops (Table 3). Gene mapping studies could help us target those particular genes to express desirable traits such as high nutritional and low antinutritional factors that can improve the genetics of both crops. Thus, the current review was undertaken to compile all the breeding studies, available germplasm, and other genetic resources to accelerate the process of genetic improvement in these crops to create varieties with low antinutrient factors and tolerance to biotic and abiotic stresses. The genetics of qualitative and quantitative characters would help breeders to adopt better breeding methods (25, 26).

## 2. Nutritional and nutraceutical qualities

**Figure 1:**
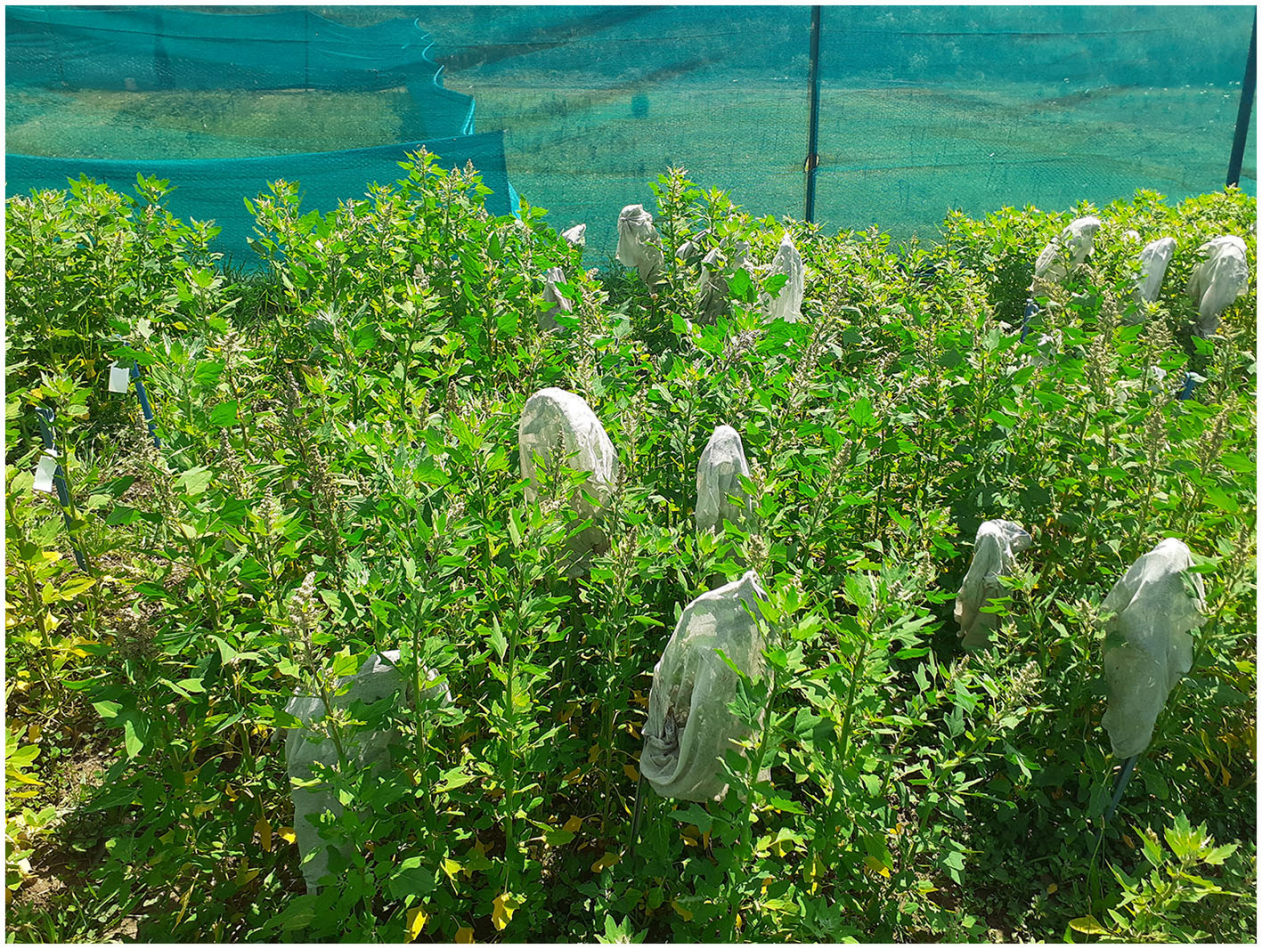
Plantation and practice of self-pollination in quinoa in Western Himalayan conditions.

Many reviews about the nutritional benefits of amaranth and quinoa showed higher protein content, higher fiber, low saturated fat, and balanced amino acid composition of their seeds than other major cereals (27, 28) (Tables 1, 2; Figure 1). The grain amaranth has many essential micronutrients such as calcium, magnesium, iron, vitamin C, β carotene, and folic acid (54, 55). The young leaves of amaranth are consumed (56, 57). The amaranth grain has great nutraceutical value and thus is known to be a new-millennium crop (58). Ripen seeds of amaranth are very famous in hills. The grain amaranths seed is high in crude protein (22.5%), dry fiber matter (8%), and high lysine content (0.73–0.84%) more than maize (3–3.5 times) and wheat (2–2.5 times) (36, 59). The amaranth seeds have mainly high methionine and lysine contents and high levels of squalene which play a precursor for all steroids. The most studied nutritional aspect concerning the food value of grain amaranth is the identification of the limiting amino acids of the protein component. Out of 20, 17 amino acids such as isoleucine, leucine, lysine, cysteine, phenylalanine, tyrosine, threonine, methionine, valine, alanine, arginine, glutamic acid, aspartic acid, glycine, histidine, proline, and serine are present in amaranth (28).

**Table 1:** Nutritional composition of grain amaranth.

**S. No**.	**Constituents**	**Type of grain species**	**Content (%)**	**References**
1.	Carbohydrates	–	65.25	(29)
2.	Fiber	–	6.7	(29)
3.	Moisture	–	11.29	(29)
4.	Protein	Yellow	13.56 14.1	(29) (30)
5.	Lipid	–	7.2	(29)
6.	Ash	–	2.88	(29)
7.	Oil content	White Red	6–10 8.5–11 11–14	(31) (32)
8.	Iron	–	0.761	(29)
9.	Zinc	–	0.287	(29)
10.	Magnesium	–	0.248	(29)
11.	Manganese	–	0.0033	(29)
12.	Potassium	–	0.508	(29)
13.	Calcium	–	0.159	(29)
14.	Polyunsaturated fatty acids	–	77 76	(33) (34)
15.	Oleic acid	Red, White	26.5–31 34 19–35	(32) (35) (34)
16.	Palmitic acid	Red, White	14–20 19 12–25	(32) (35) (34)
17.	Stearic acid	Red, White	2–3.5 3.4 2–8.6	(32) (35) (34)
18.	Linoleic acid	Red, White	32–41 50 33 0.3–2.2	(32) (33) (35) (34)
19.	Docosahexaenoic acid (DHA)	Red, White	7–21 9	(32) (35)
20.	High saponification value	Red, White	130–190	(32)
21.	Iodine value	Red, White	100–113	(32)
22.	Unsaponifiable matter	Red, White	5–7	(32)
23.	Crude protein	–	22.5	(36)
24.	Dry fiber matter	–	8	(36)
25.	Lysine content	– – Yellow	0.73–0.84 4.9–6.1 5.1–6.4	(36) (37) (30)
26.	Methionine	Yellow	0.4–1.0	(30)

**Table 2:** Nutritional composition of *Chenopodium quinoa* seeds.

**S. No**.	**Composition**	**Content**	**References**
1.	Carbohydrate (%)	69 68.84–75.82 63 55.3 69.0 74.7% 69.7 60.0	(38) (39) (40) (41) (42) (43) (44) (45)
2.	Ash (%)	3.33 13.96–15.47 3.2 3 3.8 3.2 3.0 3.7	(38) (39) (40) (41) (42) (43) (44) (45)
3.	Protein (%)	13.8 3.04–5.46 16.4 11.7 16.5 16.7 15.6 12.5	(38) (39) (40) (41) (42) (43) (44) (45)
4.	Fat (%)	5.4 4.69–6.85 6.3 12.4 6.3 5.5 7.4 8.5	(38) (39) (40) (41) (42) (43) (44) (45)
5.	Crude fiber (%)	12.88 1.92–3.38 6.3 2.2 3.8 10.5 2.9 1.92	(38) (39) (40) (41) (42) (43) (44) (45)
6.	Histidine (g/100 g)	3.2 2.0 2.7 3.1	(42) (45) (46) (43)
7.	Isoleucine (g/100 g)	4.4 7.4 3.4 3.3	(42) (45) (46) (43)
8.	Leucine (g/100 g)	6.6 7.5 6.1 5.8	(42) (45) (46) (43)
9.	Lysine (g/100 g)	6.1 4.6 5.6 6.1 6.6	(42) (45) (46) (43) (47)
10.	Methionine + cysteine (g/100 g)	4.8 4.5 4.8 2.0 2.4	(42) (45) (46) (43) (47)
11.	Phenylalanine + tyrosine (g/100 g)	7.3 7.5 6.2 6.2	(42) (45) (46) (43)
12.	Threonine (g/100 g)	3.8 3.5 3.4 2.5	(42) (45) (46) (43)
13.	Tryptophan (g/100 g)	1.2 1.1 1.1	(42) (46) (47)
14.	Valine (g/100 g)	4.5 6.0 4.2 4.0	(42) (45) (46) (43)
15.	Calcium (mg/100 g)	86.9 32.9 56.5 148.7 94 87.4 127.4 27.5 56.5 102	(48) (49) (50) (42) (46) (51) (52) (45) (53) (47)
16.	Magnesium (mg/100 g)	502 206 176 249.6 270 26 176	(48) (49) (50) (42) (46) (51) (53)
17.	Phosphorus (mg/100 g)	732 468.9 383.7 140 530 386.9 424.4 468.9 140	(48) (50) (42) (46) (51) (52) (45) (53) (47)
18.	Iron (mg/100 g)	15.0 5.5 14.0 13.2 16.8 8.1 2 2.6 1.4 10.5	(48) (49) (50) (42) (46) (51) (52) (45) (53) (47)
19.	Potassium (mg/100 g)	732 1,193.0 926.7 1,200 696.7 1,193 822.5	(48) (50) (42) (51) (52) (53) (47)
20.	Copper (mg/100 g)	5.1 3.7 1 0.2	(42) (46) (51) (53)
21.	Oleic (g/100 g)	23.3 26.0 24.8	(42) (46) (51)
22.	Linoleic acid (g/100 g)	53.1 50.2 52.3	(42) (46) (51)
23.	Linolenic acid (g/100 g)	6.2 4.8 3.9	(42) (46) (51)
24.	Thiamine (mg/100 g)	0.38 0.4	(42) (51)
25.	Riboflavin (mg/100 g)	0.39 0.2	(42) (51)
26.	Folic acid (μg/100 g)	78.1	(51)
27.	Vitamin C (μg/100 g)	16.4 4.0	(51) (42)
28.	Vitamin E (Alpha-tocopherol; mg/100 g)	5.37 2.6	(42) (51)
29.	Vit A (mg RE/100 g)	0.2	(51)
30.	Naicin (B3)	1.06	(42)
31.	Total dietary fiber	13.56–15.99	(39)
32.	Sugar content in Quinoa (g/100 g dry matter)	6.20	(39)

The unique gel characteristics of amaranth starch are also the reason for the increasing demand for amaranth (60). The starch granules in grain amaranth are polygonal, having a diameter (1–3 μm) and a very high swelling power (61). Waxy and non-waxy starch granules were present in amaranth (62). Amaranth germ contains 6%−10% of oil (31, 33, 63), mostly unsaturated oil (76%) having high linoleic acid, which is necessary for the human diet. Amaranth is a good source of energy food as it contains high protein and high-fat content, thus is a potential source of high calories. Milled and toasted amaranth products are perfect for digestion and absorption (64). Only high-protein rice is considered to satisfy protein and energy needs than other cereals (28, 65). The main constituent, amarantin substance (C_29_H_31_N_2_O_19_), is alkaloids-betalains, present in amaranths derived from grain species of amaranth (*Amaranthus caudatus* L., *Amaranthus tricolor* L., and *Amaranthus cruentus* L.) and are also used as medicine (66) and as food colorants (67, 68) having antioxidant properties (69).

Amaranth has many antinutritional factors which make it less popular to be used as major food grains. It has phytates, phenolic compounds, trypsins inhibitors, chymotrypsin inhibitors, and amylase inhibitors as unwanted constituents which need to be reduced by processing or by developing varieties having less amount of these factors (70). The phytates levels (0.52%−0.61%) were higher than in rice but much less than in corn and wheat, and tannins levels (0.043%−0.116% catechin equivalents) were much lesser than in sorghum and millet (71). Tender leaves from young Amaranth plants are consumed in Mexico and Kenya and used as an ingredient in common meals.

*Amaranthus dubius, Amaranthus viridis*, and *A. tricolor* leaves are known for their medicinal and nutraceutical properties (72). Amaranth has active ingredients that act as phytochemical compounds from its leaves (73, 74). These active components are tannins, saponins, phenols, flavonoids, cardiac glycosides, steroids, and triterpenoids (75). These chemicals have antipyretic, anti-inflammatory, antihepatotoxic, antiulcer antiallergic, and antiviral activity (75). In traditional medicines, it is used to reduce labor pain (75). In Spain, *Amaranthus* leaves and root paste is used directly on the skin to cure eczema, psoriasis, gonorrhea, menorrhagia, bruises, burns, and rashes (76). It is also used to cure urinary tract diseases, treat intestinal worms, and gastric ulcers, and as a laxative to improve digestion problems. It is good to improve appetite. It is also good for treating eye infections and breathing-related problems like asthma (75). Amaranth leaves are used as protective food due to their curative properties having strong antioxidant and phytochemical compounds present in them (73, 77, 78). If boiled leaves are given to patients suffering from jaundice, rheumatic pains, and stomach aches, it works wonders to cure them. The paste of the root is also possessing several beneficial effects when used internally and externally. The paste of roots of Amaranth controls vomiting and is thus good for dysentery patients, and the paste with black pepper is given to rabies patients (79). Its seeds can be consumed directly to stop internal bleeding, excessive menstruation, and diarrhea. It also works externally as a poultice for broken bones. The whole plant of Amaranth is also helpful in treating cholera, piles, and snake bites (80).

For centuries, *Chenopodium* spp. has been cultivated by human populations in a few parts of the world, among them few species were used as leafy vegetables (*Chenopodium album*) and a few species as grain crops (*C. quinoa* and *C. album*). It is good for the human diet due to its high protein content (14.1%) with high lysine (5.1%−6.4%) and methionine (0.4%−1.0%) contents (30, 81). *Amaranthus caudatus* is also used as animal feed. Quinoa has high fiber (7.0%), vitamins (thiamine and niacin), and minerals such as phosphorus and potassium (82–85). Due to layers of calcium oxalate on leaves, quinoa plants can tolerate droughts. As such grains cannot be consumed due to the unpleasant taste of their grains containing saponin (a glycoside), but nowadays, some types of grains are selected that no longer have that flavor (86).

Quinoa can be used instead of rice, as a hot breakfast cereal or for making baby cereal by boiling it in water. Even popping the seeds like popcorn is an option. Seeds can be sprouted or processed and used as flour. Sprouts must turn green before being put into salads (84). Ancient populations discovered the excellent nutritional content of quinoa, called it “golden grain,” and considered it an auspicious food on good occasions (87). The protein in pseudo cereals, such as quinoa, is mostly located in the endosperm. Albumin and globulins make up around 44%−77% of the protein fraction, whereas prolamins, a group of proteins associated with gluten, make up just a very small percentage of the protein fraction (0.5%−7.0%) or are completely absent in other kinds (88). Quinoa is rich in phosphorus which is five times more than cow milk and rice (89). Value additions in breads and snack food such as oats use quinoa. It may be either consumed as whole flour directly or mixed with other cereals like rice or in other recipes in combinations. Its beer can also be produced by a fermentation process similar to malt (90). *Chenopodium quinoa* is a rich source of all goodies such as minerals (calcium, magnesium, iron, phosphorus, potassium, manganese, zinc, copper, and sodium), high fiber, vitamins E and C, and vitamin B complex [such as thiamine (B1), riboflavin (B2), niacin (B3), and folic acid (B9)]. Quinoa has more nutrition than other traditional cereals such as barley, maize, rice, and wheat (14, 91–94).

The consumption of quinoa as a grain is less due to the presence of the antinutritional factor saponin in it that is present in the seed coat to protect the plant from the attack of insects and pests, but it needs to be removed before consumption (95, 96). To make quinoa worldwide staple food, we need to work on quinoa processing to reduce the saponin content of the quinoa seeds (97–101). Improved varieties had been developed called “Sweet” varieties with less saponin content but are less protected from insects, pests, and certain herbivores attack (102, 103). Many other factors such as phytic acids, tannins, and protease inhibitors are also present in quinoa. Phytic acids are present in the outer layers and the endosperm of quinoa seeds. Phytic acid binds minerals, thus it reduces the absorption of minerals in our body.

Quinoa has saponin, which acts as anticarcinogenic and hypocholesterolemic and is useful for health (49, 104). Polyphenols are present in quinoa and there are three main polyphenols (flavonoids, phenolic acids, and tannins) which are the reason for bitterness, astringency, color, taste, and oxidative stability (105–107) and also act as an antioxidant thus preventing cardiovascular diseases (39), anti-allergic, anti-inflammatory, antiviral, and anticarcinogenic. In ancient times, black quinoa was mixed with alcohol and applied to the wounded area. Quinoa grains are used by patients suffering from muscle sprains, twists, and muscular strains (108).

The domestication of amaranth declined at the time of Spanish arrival, but the reasons for the decline of this crop are unclear (109). Many myths explained the decrease in grain amaranth cultivation. Maize coevolved with amaranth, but now maize is a major cereal crop and this crop remains underrated despite its more health benefits, and it is a more nutritionally valuable food for the human population. The size of the seeds of amaranth may be the reason for the reduction in cultivation of this crop as compared to maize. Small-seeded crops require more care in handling than larger-seeded crops. The cultivation of amaranth becomes a part of small plots only in Mexico, the Andean region, and a few areas of India, Bhutan, and Nepal (110). Currently, it is cultivated throughout Asian countries such as China, Bhutan, India, Indonesia, Nepal, Malaysia, Philippines, Central America, Mexico, and Southern and Eastern Africa. Another reason might be the beauty of amaranth leaves which prevent this crop from disappearing from the world as it was always part of the garden in rural areas and is fascinating and difficult to ignore. America leads in quinoa production. Due to crop diversification, quinoa production increased by 72% in Peru, Bolivia, and Ecuador (84).

## 3. Plant descriptors

### 3.1. Amaranth

**Figure 2:**
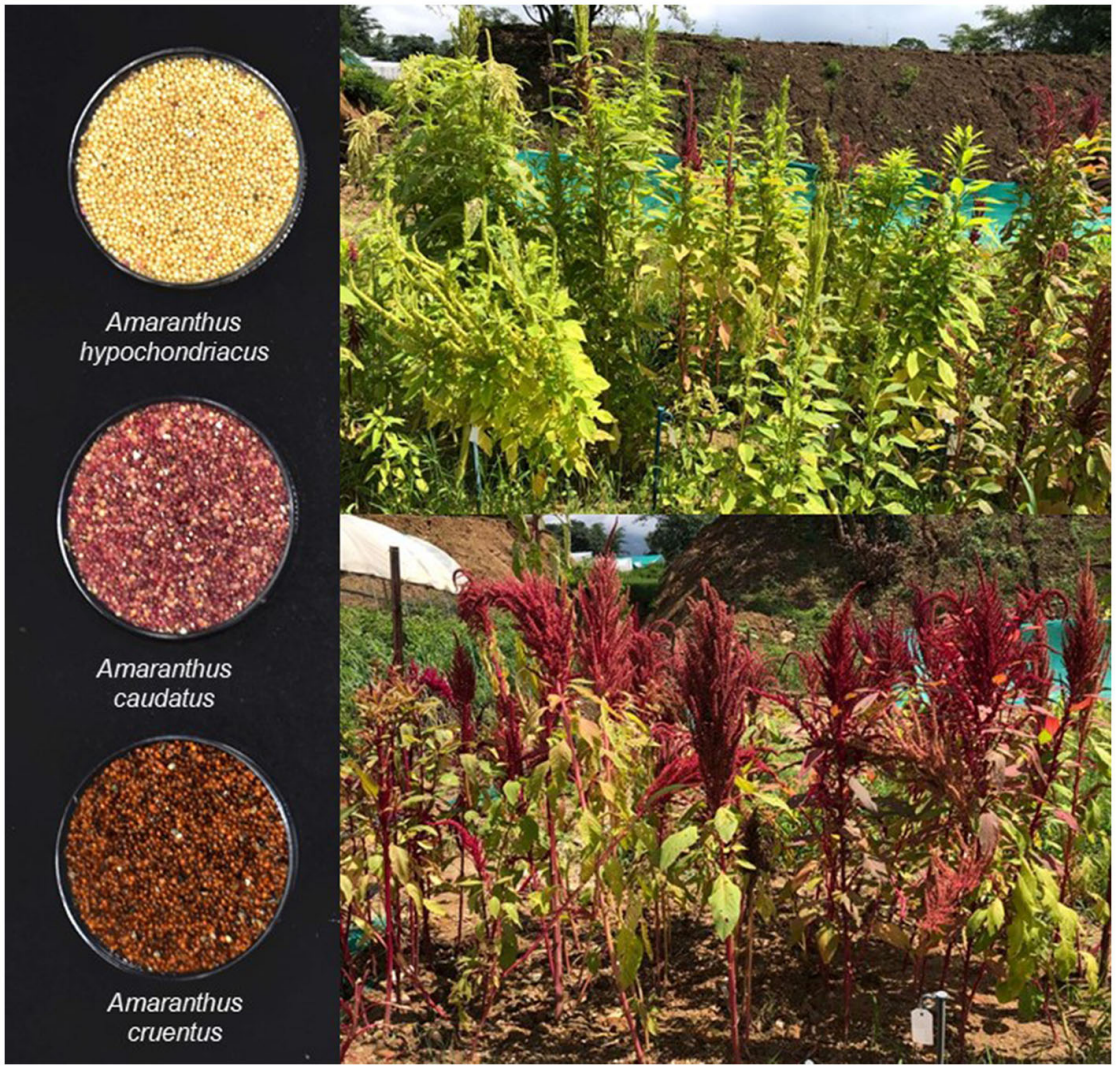
Variation among grain amaranth species.

Amaranth is a non-grass annual plant, having an herbaceous stem with height varying from 30 to 210 cm, a solid stem of varying colors, petioled morphology, ovate in shape, hairy or non-hairy with wavy margins and alternate pattern of length (7.5–15 cm), and different colored leaves. The size of the flower varies from 1 to 2 cm in different colors from red to maroon. The seeds are oval in shape with colors, white, red, and black, that germinate at high humidity (111). Amaranth seeds have folded flange, reticulation cartridges over spermoderm, and the presence of verucate processes, and fruits have dehiscent pyxis (112). It is having taproot system which is long, fleshy red or pink, deep, and developed, thus it stands in water stress conditions. Amaranth is monoecious, with few dioecious species. Pollination occurs by wind or by insects. The color of amaranth leaves varies from bright red to violet, and its maroon color is due to battalions pigments (6). Its inflorescences are 0.50–0.90 m long and glomerulated (Figure 2), erect or inclined, and yellowish, reddish, or purple in color.

The phyllotaxy and vascular supply study was carried out in *A. caudatus* (113), *Amaranthus graecizans* L., and *Amaranthus hybridus* L. (114), and in other common species by Costea and Demason (115). Cultivated grain amaranth (*A. caudatus, A. cruentu*s, and *Amaranthus hypochondriacus*) from their wild ancestors were studied for segregation within the “hybridus complex” with characters related to morphology, phyllotaxy of leaves, epidermal characters of leaves, vascular supply studies, and secondary growth in plants.

Amaranthus's pollen grains are pantoporate with more than 18 sunken pores and tectate with granules and spinules (116, 117). Similar type pollens were present in the other members of Amaranthaceae (118) and several other Centrospermous families (119). Amaranth has different ploidy levels and interspecific hybrids determined by applying pollen grain features. In dioecious species, pollen grains have multiple apertures on the surface (120). The size of pollen grains is dependent on the ploidy level of the plant. The lower ploidy level has a smaller size, size increases with an increase in the ploidy level. In polyploids, the exine shows more patterns than in diploids (121).

### 3.2. Quinoa

Quinoa is a dicotyledonous annual plant belonging to the Amaranthaceae family. The plant grows well in India, with several varieties having a height of 1.5 m, with a number of branches, and a large leaf size (122). There is a well-developed ramified tap root system protecting against drought conditions (123). The seeds are round and flat, have a seed diameter of 1.5–4.0 mm, and take a variety of forms from white to gray and black, with shades of yellow, rose, red, purple, and violet. Unisexual female flowers are a significant characteristic of quinoa (124, 125). Quinoa has three panical shapes: amarantiform, intermediate, and glomerulate; amarantiform ones have elongated glomeruli growing from the secondary axis, while the others have spherical glomeruli growing from the tertiary axis and sometimes a plant will display both traits, producing an intermediate inflorescence (126). Gandarillas (127) also noted that a dominant allele influences the glomerulated panicle feature. The ovary is superior having two or three stigmas, five perianth lobes, and five anthers. Some cultivars exhibit partial or complete male sterility in the female flowers. According to Risi and Galwey (128), the fruit (achene type) can be conical, cylindrical, or ellipsoidal with three layers present: perigonium, pericarp, and episperm, with saponins present in the pericarp. Seeds vary in size and color, with black dominance over both colors and red dominance over white (128). Quinoa has shown good tolerance to varied temperatures in both temperate and tropical regions. Prego et al. (129) found that quinoa seeds' perisperm, embryo, and endosperm contain nutritional reserves. In some places, such as the Andean highlands, planting season lasts from December to January; in others, it starts in August and lasts until December. Where mechanized agriculture techniques are utilized, quinoa is planted in row spacing (40–80 cm) (84).

## 4. Genetic resources

### 4.1. Amaranth

Amaranth has a basic chromosome number (*x* = 8 or 9) (130) and is allotetraploid with chromosome number *n* = 16 or 17. The first domesticated species (*A. cruentu*s) originated from *A. hybridus* in Central America; then, *A. hypochondriacus* by recurrent crossing between *A. cruentus* and *A. powellii* in Mexico and the domestication of *A. caudatus* by crossing between *A. cruentus* and *Amaranthus quitensis*. These three grain *Amaranthus* species along with *A. hybridus* formed a complex or aggregate (“hybridus complex”) structure.

Amaranth is an ancient crop belonging to the Amaranthaceae family, Caryophyllales order, and Amaranthoideae subfamily. The genus *Amaranthus* has ~87 species and out of them 17 are from Europe, 14 are from Australia, and 56 are from America (87, 131, 132). Moreover, out of 87 identified species of amaranth, 17 species are for vegetative purposes, and mainly three for grain purposes, namely, *A. caudatus, A. cruentus*, and *A. hypochondriacus* (133), and the others are widely dispersed weeds. All these grain amaranths are famous for their magnificent appearance and are popularly known by a few names, such as *A. hypochondriacus*, famous as prince's feather, *A. cruentus* as purple amaranth, *A. caudatus* as love-lies-bleeding, grown more as an ornamental, and *A. tricolor* as tampala, grown mostly for the leaves. Among them, *A. caudatus* is mainly a tropical plant species. Other vegetable species are *A. dubius, Amaranthus blitum*, and *A. cruentus*; weed species are *Amaranthus retroflexus* (as redroot pigweed), *Amaranthus albus* (and also tumbleweed), and *Amaranthus spinosus* (due to spiny leaves as spiny amaranth). Amaranth species within themselves have fewer genetic differences, thus different species can do easy crossing over and even wild types will cross with domesticated varieties if not timely rogued from the field (83).

*Amaranthus cruentus* accessions (“African” grain type) have seeds of dark colors which branched heavenly belonging to West Africa and have inflorescence which matures early. The other accession, *A. hybridus* L. (“Prima” grain type), is also a dark-seeded, highly branched, short plant stature belonging to Asia. Few barriers were reported to prevent inter-specific hybridization between crosses of *A. cruentus L., A. hypochondriacus* L., and *A. hybridus* L. Like, whenever hybridization of *A. caudatus* L. and with any grain type was carried out, this often results in non-viable seed formation (134). Adaptations and migration patterns were predicted for all amaranth species and showed potential for adaptation to diverse climate regions because it showed a wider adaptation rate than other wild and cultivated species (135). About 121 different crops and wild individuals of amaranth were studied through a relative genetic map and analyzed that grain amaranth was domesticated from a single wild ancestor (136).

The germplasm of Amaranth is widely spread across the globe including the USA, India, and Peru. In the USA, they are grown for healthy diet food while in countries such as Peru, Bolivia, India, and Mexico, it is a traditional food that lost its identity after the introduction of new world cereals such as wheat and rice (132). The USA germplasm collection has a conservatory of 3,300 accessions of *A. hypochondriacus* from 40 different countries (137). In India at the National Bureau of Plant Genetic Resources (NBPGR), there is a collection of 3,081 accessions (138) and the National Botanical Research Institute (NBRI) has 2,500 accessions (139) of *A. hypochondriacus*. In Peru, the Univ. Nacional San AntonioAbad del Cusco (UNSAAC/CICA) has 740 accessions of *A. caudatus* (140). Analysis of 20 accessions of amaranth species (*Amaranthus* L.) provides evidence that wild accessions from Central Malawi have more nutrients, minerals, and vitamins than those domesticated from other agro-ecological regions (141). Amaranth species accessions were assessed by using simple sequence repeat markers and genetic variability was also present (142), thus it shows the scope of variations for future breeding programs for developing a rich germplasm pool of amaranth crop. Genotypes were grouped into 10 clusters which can be selected for the hybridization program as parents, as those analyzed accessions showed variability for improvement in crop (143). Ninety-eight genotypes were analyzed for 14 characters of grain amaranth (*A. hypochondriacus* L.) for studying the relationship between genetic divergence and eco-geographical region. However, no significant results were obtained among clustering patterns (144). These clusters could be used in a hybridization program to select good parents having maximum variability and select transgressive segregants from the population developed to increase the yield. Phenotypic characterization of amaranth genotypes based on biomass yield and related traits laid the future for trait-focused breeding programs (145). Many species of amaranth were studied for the complete chloroplast genome sequences using simple sequence repeats by Chaney et al. (146). SNPs and indels proved to be great genetic resources for studying phylogeny from diverse genetic diversity. From three grain species of amaranth, 37 accessions from Nigeria were studied, and after evaluation, clusters were made (147) and concluded a great scope of genetic diversity to improve yield parameters through breeding. From 20 *Amaranthus* species, 229 genotypes were evaluated for diversity among genotypes for the improvement of high-yielding cultivars according to the origin and the preferred area of production (148). Thirty-two *Amaranthus* species were evaluated for 16 traits for the morphological characterization of genetic resources for breeding purposes (149); 13 genotypes of different species of *A. hypochondriacus* and *A. tricolor* were grouped into two major clusters, to differentiate between ornamental and edible (150). Two selected mutant lines developed through gamma radiation treatment of *A. cruentus* L. were evaluated (151), and treated plants showed significantly higher seed yield and seed weight than non-treated plants.

### 4.2. Quinoa

There are many *ex situ* conservatories for quinoa in gene banks that use seed properties to conserve germplasm. All over the world, 30 countries conserve quinoa in 59 gene banks. The *ex situ* conservatories of *Chenopodium* have 16,263 accessions preserved in the world, originated and thus maintained in the Andean Region (mainly in Bolivia and Peru) (152). The different countries that contribute to quinoa germplasm conservation are America having 10 countries associated with it, Europe having 11 countries, Africa having five countries, and Asia having three countries. Bolivia and Peru have the largest diverse regions of all. In Bolivia, there are 6,721 quinoa accessions, Peru has 6,302 accessions, Argentina has 492 accessions, Ecuador has 673 accessions, Chile has 286 accessions, and Colombia has 28 accessions. These conservatories are run by different institutes all over the world, such as INIAF (Instituto Nacional de Innovación Agropecuaria y Forestal—National Institute of Agricultural and Forestry Innovation), UMSA (Universidad Mayor de San Andrés—Major University of San Andrés), UTO (Universidad Técnica de Oruro—Oruro Technical University), UCB (Universidad Católica Boliviana—Bolivian Catholic University), UPEA, and in the Centro de Investigación y Promoción Comunal (Municipal Research and Promotion Center—CIPROCOM) (153). Seed collections conservatories are also in South America at Universidad Nacional del Altiplano (UNAP, Peru), the National Institute of Agricultural Research (INIA, Peru), the Research Center for Andean Studies (CICA, Peru), the National Seed Bank of Chile, Royal Botanical Gardens Kew (UK), the USDA-ARS (USA), the National Bureau of Plant Genetic Resources (India), and IPK-Gatersleben (Germany) (154). Wild *Chenopodium* species have 357 accessions from USDA-ARS and the Royal Botanical Gardens Kew (132). Quinoa has two distinct germplasm pools: Andean highland quinoa, which is the primary germplasm, and central and southern Chilean quinoa, the second germplasm center (155), and Argentina of *C. hircinum*, the third germplasm pool, which are major areas from South America (156). The classification is done based on different agro-morphological variables such as growth habits (four growth habits), plant color (panicle emergence or start of flowering), panicle shape and density (amarantiform or glomerulate or intermediate), grain color and shape (white, cream, yellow, orange, pink, red, purple, light coffee, greenish coffee, or black), grain size (ranges from 1.36 to 2.66 mm), crop cycle (physiological maturity within 119–220 days), and grain yield (13).

## 5. Breeding behavior and approaches

### 5.1. Genetic improvement of amaranth

Amaranth is a self-pollinating crop, with few percentages of cross-pollination (157). Amaranth is monoecious in nature. Homozygous, true-to-type lines were maintained by repeated self-pollination and within selection for 6–8 generations and further used for hybrid production. For hybridization, male and female parents are planted at a proper isolation distance. Emasculation procedures and controlled pollination were performed and developed (158, 159). Chromosomal studies were carried out (160, 161) and polyploids were studied (162). Indian germplasm showed a lot of diversity as documented by researchers (163). The mode of inheritance of traits was studied in amaranth crop and inheritance was studied for different characters (164, 165), including yield parameters (166). Parameters such as harvest index and grain yield were studied (167).

Inheritance studies were important for improved hybrid production for selecting important characters which are significant to be carried out further. Gene markers are the easiest approach to studying the inheritance of desirable traits for developing good varieties. Markers for a few parameters such as plant growth, plant morphology, seed characteristics, and flowering behavior were identified in the past, and their inheritance was studied thoroughly (168–170). Inheritance of nutritional facts, including starch characteristics and perisperm layer of grain, was studied in Japan (62, 171, 172). Studies were also carried out in India about seed protein content and their inheritance (173). Inheritance of male sterility was investigated and used for hybridization (174, 175).

Good hybrids are produced by selecting germplasm which is rich in desirable characteristics. Hybrids' performance depends only on carefully selecting good recombinants from the present gene pool and combining them intelligently with each other. Selection is made according to environmental adaptations and beautiful vigorous vegetative features with respect to growth and yield characteristics. The environment affects the expression of many traits such as plant height, days to maturity, and plant habit. Interspecific hybridization is carried out successfully between a few species, and crosses between the species *A. cruetus L., A. hypochondriacus* L., and *A. hybridus* L. produce viable offspring (134). Few interspecific hybrids were successful on a commercial scale also. In the United States, a large area was under the cross of *A. hypochondriacus* and *A. hybridus*; the amaranth grain production guide was developed to study agronomical methods to grow these hybrids (176). Genetic stability has been achieved for the development of improved lines by interspecific hybridization. Amaranth plants have undergone improvements for many decades using hybridization, selection, and mutagenesis methods.

Genetic diversity research is essential for utilizing plant genetic resources for amaranth crops (8, 177). Amaranth genotypes also show evolution by the influence of the environment of specific agro-ecological regions (178). Introgression and hybridization between species showed great variability and phenotypic plasticity (179). Mass selection and pure selection methods were used to improve the amaranth germplasm by self-pollination and cross-pollination methods (169, 180, 181). *Amaranthus hypochondriacus, A. caudatus*, and *A. cruentus* species were cream-colored seeds generally used for grain purposes. These present-day species were domesticated from the wild black-seeded *A. hybridus* (132, 182). Studies had been conducted to study genetic diversity among Indian populations of amaranth (163). Different breeding strategies were used to study different parameters in amaranth (164–166).

The study of genetic control of trait(s) is a basic requirement for the purposeful management of genetic variability. Both additive and non-additive gene effects are recorded for different parameters in grain amaranth (167, 183). Multiplicative characters such as yield or panicles plant^−1^ are controlled either predominantly by non-additive or larger non-additive than additive components (167, 183). Research on heritability and genetic advances are reported to be mostly moderate to low for yield plant^−1^, panicles plant^−1^, panicle length, seed weight panicle^−1^, and test weight (184). Plant growth, grain harvest index, days to 50% flowering, and days to maturity were observed to exhibit largely moderate-to-high heritability and genetic advance. The maximum genetic gain would be difficult to realize by exercising selection on seed yield or panicle alone. Selection based on yield components with more weightage on panicles per plant and grain weight per panicle would be more beneficial (184). A recurrent selection program for the exploitation of both of these gene actions would be useful in grain amaranth as both additive and non-additive gene effects controlled agronomical traits. Additive gene variance and enhanced genetic recombination were used for the diallel selective mating system. Considerable heterosis has been recorded in grain amaranth. Pandey (167) reported heterosis over better parents to 71.36% for grain yield per plant. The manifestation of heterosis for grain yield per plant seemed to be primarily due to the heterotic response of panicle per plant. Sterile lines can be developed for the production of hybrids seed, but till now, no male sterile line is identified for the commercial scale for seed productions.

To reconstitute the plant for creating genetic variation, improving adaptability, imparting resistance, alleviating nutritional status, and developing ideal plant types, several attempts are being made taking the recourse of polyploidy and hybridization at the interspecific level. Autotetraploids are developed in several species/varieties of grain amaranth (134, 185). Pal and Khoshoo (134) raised autotetraploid in *A. hypochondriacus* var. AG-21 and with normal growth that produced bold seeds but suffered with the reduction of grain yield plant-1 perhaps due to the reduction in the number of seeds glomerule. Pal and Pandey (186) again induced autotetraploidy in several grain amaranth species but observed a similar trend. This indicates that polyploidy does not hold well in this crop but such variation can be used for further breeding programs. Scientists are trying to raise allopolyploids from species hybrids between the wild/weedy and cultivated types as well as at the intraspecific level (162, 186). Pal and Khoshoo (134), Khoshoo and Pal (162), Pal (187), Pal et al. (188), and Pal and Pandey (186) produced both wild and cultivated species of grain amaranth. These interspecific hybrids usually showed high variation in pollen and seed fertility. Some interspecific combinations suffered from sterility. But, in general, the hybrids were fertile and there was copious seed formation. Thus, the desirable features of wild/weedy species can be introduced into the edible types of grain amaranth. Future research can be focused on the development of embryo culture, anther culture, and gene transfer.

In recent years, the plant's chemical composition and agronomic properties have improved using biotechnological methods. Genetic engineering methods make it possible to improve not only the useful properties of a plant but also to provide additional useful characteristics during plant transformation. Different technologies such as *Agrobacterium* transformation and direct gene insertion by biolistic gene gun transfer methods insert genes with the desired gene. Genomic-assisted breeding (GAB) is very helpful to select potent parents with novel characters for breeding using hybridization and selection in future. Studies such as chloroplast gene sequencing, genetic mapping, and the use of biomarkers are useful to make easy the task of choice combinations to carry out breeding approaches. Chloroplast genome assembly of *A. hypochondriacus* (Amaranthaceae) was developed (146) to reconstruct the chloroplast genomes between two related grain species (*A. cruentus* and *A. caudatus*) and their putative progenitor (*A. hybridus*). Lightfoot et al. (189) identified a major QTL for flower color in *A*. *hypochondriacus* using linkage mapping. The physical map of the *A*. *hypochondriacus* genome has 16 chromosome-scale major scaffolds with an N50 of 24.4 Mb (189). Germplasm from *A*. *hypochondriacus* species (22) and SNP alleles were generated and a genome-wide association study (GWAS) was carried out for the qualitative traits between specific phenotypes and genetic variants within the genome and identified marker-trait associations (MTAs) on 16 amaranthus species. SNP markers were used to produce genetic resources for the phenotyping and development of cultivars. Genetic similarities were also important for the effective use of available germplasm by using phenotypic and molecular markers. Amaranth accessions were assessed for variations using random amplified polymorphic DNA (RAPD) primers (24) for getting information on genetic diversity for reliable gene recombination. For carrying out a successful hybrid program, analysis of phenotypic diversity and traits is very valuable.

Taxonomic confusion exists among *A. hybridus* species complex, among all species (*A. cruentus, A. caudatus, A. hypochondriacus, A. hybridus, A. quitensis*, and *Amaranthus powellii*) (190). DNA markers were used to examine the taxonomic and phylogenetic relationships of grain amaranth and their wild relatives and to study phylogeny.

### 5.2. Genetic improvement of quinoa

**Figure 3:**
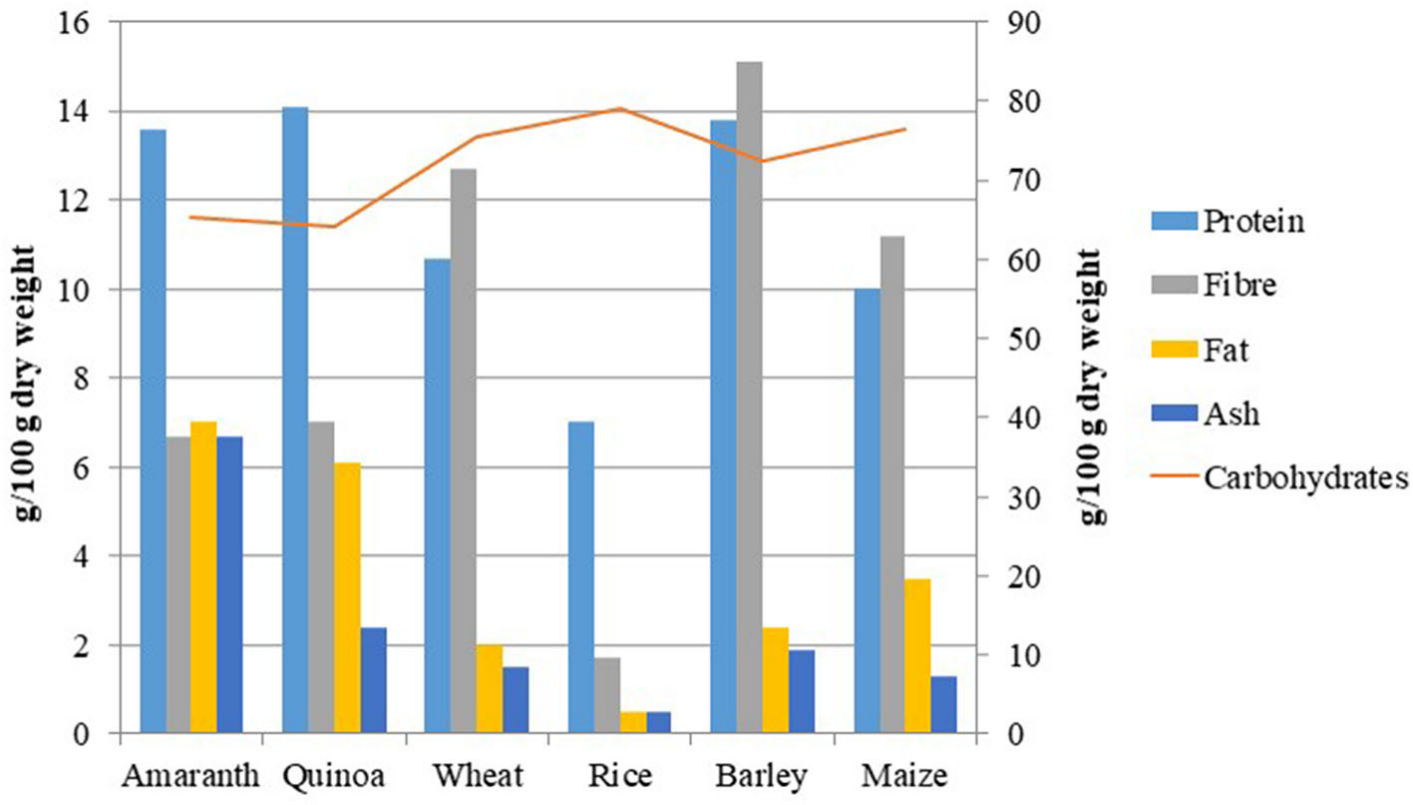
Comparison of the general nutritional profile of amaranth and quinoa with major cereals (28).

Quinoa is a self-pollinated crop with few crossing chances (17.36%) and purity can be maintained by enforced selfing by covering the inflorescence stalk with a paper bag (Figure 3) (191). Cleistogamy leads to autogamy in quinoa; however, obligatory out-crossing due to self-incompatibility and male sterility have been reported (192). The breeding objectives for quinoa were the short stature of plants with fewer branches, growth, short cycle, earliness, low saponin content abiotic and biotic stress, and higher grain yields for commercial varieties by mass selection and hybridization for direct future breeding strategies. The diversity present in the primary gene pool help to characterize Quinoa (128). Due to Quinoa's extensive genetic variety, wide agronomic adaptability, tolerance to many soil types, notably salty soils and situations with highly varying humidity, elevations, and temperatures, and good nutritional health benefits, breeding studies for Quinoa are important (193). To fulfill farmers' demands in various varied environments and agronomic systems, global collaborative work is needed for quinoa which might serve as a basis for improved plant breeding initiatives for developed or developing nations.

DNA markers and linkage maps are important tools for germplasm conservation and crop improvement programs. The first molecular studies were using allozyme markers to establish genetic variability in domesticated quinoa and wild species (*C. hircinum* and wild quinoa ajara) (194, 195). The distribution of quinoa is high in the Andes in the context of climate changes affected by drought and frost and managed based on the latest technologies and the ecosystem (196). Quinoa has two distinctive groups: a coastal type from southwestern Chile and an Andean type from northwestern Argentina to southern Colombia and their co-evolutionary relationship between domesticated and wild populations is based on molecular studies (195). Quinoa is characterized based on seed storage proteins for the identification of cultivars and breeding purpose for improved protein quantity and quality (197). Morphological variability and various selection parameters were assessed in 44 germplasm lines of *Chenopodium* spp. (198). Taxonomical and biochemical studies and cross-ability relationships were reported on these species in quinoa (199).

Genetic variability is important for rich germplasm and their conservation for genetic improvement in quinoa. Furthermore, molecular markers are very beneficial in allowing the development of better breeding programs. Genetic resources are very helpful in quinoa cultivars improvement using marker-assisted selection and marker-aided backcross breeding.

As DNA markers, random amplified polymorphic DNA (RAPD) was used first time in quinoa by Fairbanks et al. (200). These markers detect genetic variations within the existing germplasm collection (201, 202). Genetic linkage maps could be generated from intergeneric crosses for hybrid production by using DNA markers (203). Different types of markers such as RAPD and ISSR were used for genetic variation by molecular analysis in quinoa. These markers play a very significant role in distinguishing different genotypes. Simple sequence repeat (SSR) markers were also helpful due to their co-dominant effect and their capability to detect polymorphism in quinoa (204). Various researchers have also studied the effect of SSR markers in different crops and also in quinoa. Jarvis et al. (203) developed 216 new polymorphic SSR (simple sequence repeats) markers from libraries enriched for GA, CAA, and AAT repeats, as well as six SSR markers from bacterial artificial chromosome-end sequences (BES-SSRs) in quinoa. Maughan et al. (205) also reported that the primer sequences and map locations for 19 SSR markers are valuable tools for quinoa genome analysis. This map also indicates starting point for genetic dissection of quinoa, grain yield, seed saponin, maturity, and resistance to diseases, frost, and rot. Fluorescence-tagged markers are used to characterize genetic diversity within quinoa germplasm. Microsatellite markers were used for the characterization of *C. quinoa* (206). These markers showed genetic diversity between quinoa and other *Chenopodium* species (207). Microsatellite markers confirmed high polymorphism between two and three nucleotide motifs and the differences in having repeats of motifs (208). Single nucleotide polymorphism (SNPs) was identified by floral expressed sequenced tag (EST) libraries in the development of immature seeds in quinoa (209). This helps to understand the gene associated with its expression and regulation for seed development and gene mapping for breeding development and their evolutionary studies within the genus. The new polymorphic simple sequence repeats (SSR) markers were developed from repeats in gene libraries and few markers were created from bacterial artificial chromosome-end sequences (BES-SSRs) (203). For several marker loci, segregation distortion was observed with markers having an easily transferable nature and showed chromosomal regions linked with selection or gametophytic lethality.

The SNP assays were detected using the Fluidigm dynamic array based on KASPar genotyping (23). The SNPs are important genomic tools for molecular analysis for the improvement of desirable parameters in quinoa. Genome characterization and evaluation are important for selection and cultivar improvement (210). DNA fingerprinting is one of the best tools to assess the chromosomal studies of quinoa genotypes. Biochemical and genetic studies play a more significant role in the development of core collections than the old breeding techniques. The Flowering Locus T-Like genes were used as markers to characterize the genome of diploid species of quinoa (211). Gene editing is useful for the evolution of quinoa germplasm by inserting mitochondrial DNA in *C. quinoa*. Phylogenetic trees could also be constructed to study unknown ancestry, their relationships with each other, and the role of parents in the origin of present quinoa genotypes. Quinoa is a potential pseudocereal crop thus the study of its ancestry is important to understand sources of heat and biotic tolerance genotypes. Polymorphism was studied in quinoa accessions by using EST-SSR markers for molecular analysis (212).

Due to cross pollination, existing of heterozygosity, and allotetraploidy with genome complexity the molecular analysis of quinoa is restricted (213). To avoid such barriers, inbred lines were developed. The mechanisms involving allotetraploidy were studied for genetic evolution in quinoa. Molecular real-time sequencing with optical, chromosome-contact, and mapping studies was conducted to have a thorough view of the chromosome-scale reference genome sequence for quinoa breeding (96). The sequencing helped us to study the ancestral gene pools which allow for the characterization of sub-genomes in quinoa. The genome sequence was helpful to study the protein synthesis process for the production of anti-nutritional factors (saponins) found in quinoa and to recognize mutations affecting translation in sweet quinoa strains. Thus, quinoa is a model plant for studying polyploidy, genome evolution, and the science behind abiotic stress tolerance, especially salinity tolerance. A complete genome sequencing showed a high number of polymorphic single nucleotide polymorphism (SNP) loci which have been utilized with repeated multiple-year phenotypic data to identify QTLs (quantitative trait loci), controlling all important yield and quality parameters (214).

To expose the phylogeny, the complete chloroplast (cp) DNA was analyzed by next-generation sequencing (complete plastid genome sequences) and molecular markers (215). With sequencing, the genetic diversity of the *Chenopodium* genus was studied thoroughly. The coding and non-coding regions were analyzed. Inter-simple sequence repeat (ISSR) markers are easier to use than simple sequence repeat (SSR) markers, which are frequently used for genotyping quinoa. ISSR markers are cheap, scoring can be done manually, and a sequence of flanking sites is not necessary, but this is not the situation in SSR markers (216). Whole-genome re-sequencing, using InDel (insertion/deletion), would significantly provide information on population diversity and their role in creating future desirable genotypes in quinoa (217) and can be used to re-sequenced quinoa accessions. These markers clearly depict the difference between the Andean highland type and the Chilean coastal type quinoa.

The molecular markers were used in China to investigate variations in their varieties (218). The ISSR markers were used to recognize polymorphism and identify unique markers for genotypes. ISSR markers characterized five quinoa genotypes and they showed polymorphism (219). ISSR markers are helpful in DNA fingerprinting, and bands formed by these markers are useful to go through the phylogenetic relationship among quinoa present to cultivate wild species, thus this study guides us to identify desirable genes and their sources to plan future breeding programs. This practice also helped us to understand polymorphism in the quinoa genotypes to distinguish them from each other. Quinoa improvement could be possible by germplasm preservation, and conservation is important for developing better-performing hybrids. Molecular characterization was done by different molecular markers, i.e., ISSR, SCoT, and DNA chloroplast markers (rbcL and rpoC1), to study genetic polymorphism and to develop unique markers for each of the seven quinoa genotypes different in seed color and origin (220). These sequences provide good genetic information to the gene bank for the conservation of germplasm.

Accessions of *C. quinoa* and *C. album* were evaluated (221) and SDS–PAGE protein profiling of soluble proteins seed in the quinoa was done. Using microsatellite markers with available genome sequences, a thorough analysis of quinoa's genome and its relatives was carried out in five Amaranthaceae species (222). The results demonstrated that high proportions of nucleotide characterized the microsatellite repeats with A/T rich motifs and showed the conservation of genomes and composition of microsatellites in the Amaranthaceae family. The microsatellite markers (SSRs) were used for DNA analysis of nine loci located in 26 varieties of quinoa (223) for the genetic characterization of those varieties.

There are two ways to generate crops that are resistant to salt and drought stress. First, by adding genes from tolerant plants, main crops may be enhanced. Mostly, all the important crops have wild species which has more potential to resist drought and salinity than the cultivated genotypes. We need to understand the mechanism of the fundamental resistance processes going on within plants, then crops might be genetically modified to have higher tolerance. Second, certain marginally grown minor (orphan) crops are already salt and drought resistant. Gene editing about tolerance in crops and improving these crops' agronomic performance may be a useful strategy for boosting crop and food variety. Developing a nutrient-dense, high-performing crop that can fulfill upcoming food demand in a changing environment by selectively altering a few of these genes through induced mutagenesis appears promising. DNA manipulation will provide great tools to study key genes' role in stress tolerance regulation (87, 224, 225). There is a need to study transformation systems in quinoa to conduct gene editing research. Two transformation systems using hairy root and leaf agroinfiltration were developed for quinoa to study the genomics of quinoa, and translation in plants in transgenic quinoa roots was obtained successfully by *in vivo* method, but having low efficiency. QTL mapping of agronomic parameters can be useful for photoperiod and involved in flowering time by transcriptome analysis to identify genes differentially responding; and by contributing to the development of an Agrobacterium-mediated transformation protocol in quinoa can contribute to improving the quality of crop (226). *In vitro* studies are very important for supporting genetic engineering studies. Hypocotyl tissue proved to be a suitable explant for the micropropagation of quinoa through the induction of indirect organogenesis (227).

Traditional breeding methods take so much time (nearly 10–12 years) to develop good varieties. To accelerate the genetic improvement of these pseudocereals, we can also go for speed breeding like other major crops to reduce the time period of good quality germplasm development. In this, plants are grown in controlled conditions with controlled photoperiods, humidity, and temperature conditions. This speeds up the crop cycles of these crops so that we can get 5–6 crops in 1 year (136). The tissue culture studies allow us to grow plants throughout the year; thus, we are not dependent on the season to conduct our experiments. The studies on transformation and tissue culture can help us to conduct genetic engineering technologies to improve the germplasm of these pseudocereals (Table 3) (225, 226, 255, 258). A list of available advanced molecular and biotechnological tools and techniques is given in Table 3.

**Table 3:** Advanced biotechnological tools and techniques available in amaranth and quinoa.

**S. No**.	**Genetic material**	**Technology**	**Type**	**Purpose**	**References**
1.	*Amaranthus caudatus* L., (PI490458, AMES15114, AMES5461), *Amaranthus cruentus* L. (434, 622, AMES2248, AMES2247, PI511731, PI777913), *Amaranthus hybridus* L. (1047), *Amaranthus hypochondriacus* L. (1221, 718, 674, 722, 412, PI540446)	Tissue culture	MS_30_ media is most effective with 2.7 μM NAA+ 2.5 Mm 2ip. 2.7 μM NAA + 2.3 μM KIN, 2.7 Mm NAA + 4.4 Mm BA by using seeds as explant	To study morphogenesis and growth of calluses	(228)
2.	*Amaranthus hypochondriacus* L., *Amaranthus cruentus* L., *Amaranthus tricholor* L.	Tissue culture	B_5_ + 0.1 mg/L + 0.1–1.0 mg/L ZEA by using hypocotyles as explant	Regeneration	(229)
3.	*Amaranthus cruentus* L. “Ficha” and *Amaranthus hybridus* “K-433”	Tissue culture	MS_30_ + 5 mg/L + 0.01 mg/L NAA on explants (epicotyl, hypocotyls, root and leaf segments)	Propagation	(230)
4.	*Amaranthus* spp.	Tissue culture	MS media + vitamins + 3–10 mg/L 2,4-dichlorophenoxyacetic acid (2,4-D) + 0.05 mg/L kinetin on hypocotyl segments prepared from aseptically germinated seeds	Micropropagation	(231)
5.	*Amaranthus hypohondriacus* L., *Amaranthus cruentus* L., *Amaranthus tricolor* L.	Tissue culture	MS _30_ + 2 mg/L NAA + 0.2 mg/L BA + 10% coconut water on hypocotyles and leaf disc as explants	Regeneration	(232)
6.	*Amaranthus caudatus, Amaranthus gangeticus, Amaranthus hypochondriacus, Amaranthus retroflexus and Amaranthus viridus*	Tissue culture	MS salts + 0.01 mg/L NAA using shoot tips	Regeneration	(233)
7.	*Amaranthus caudatus* and *Amaranthus hypochondriacus*	Tissue culture	MS _30_ + 0.3 mg/L IAA + 3 mg/L KIN using hypocotyls as explant	Regeneration	(234)
8.	*Amaranthus*. *paniculatus* L.	Tissue culture	MS30 + 8–15 mg/L KIN or 5–10 mg/L BA, MS30 + 0.5–10 mg/L 2,4- D + 0.5–10 mg/L NAA using inflorescene as explant	Regeneration	(235)
9.	*Amaranthus paniculatus* L	Tissue culture	B_5_ KIN (0.5 ppm) and NAA (0.1 ppm), B_5_ + 1 mg/L GA3 (gibberellic acid) + 1 mg/L KIN + 1 mg/L 2,4-D on hypocotyls	Regeneration ability and callus formation	(236)
10.	*Amaranthus hypochondriacus* L. cv. Azteca	Tissue culture	MS_30_ + 13.2 μM BA + 1.08 μM NAA on epicotyl and hypocotyls on 7 days seedlings	Regeneration	(237)
11.	*Amaranthus tricolor*	Tissue culture	MS_30_ + 13.2 μM BA +1.8 μM NAA on 7 days seedling on epicotyl and hypocotyls part	Regeneration	(238)
12.	*Amaranthus caudatus*	Tissue culture	MS + 2.0 mg/L 2,4-D + 0.75 mg/L KIN on Callus cultures from hypocotyls and cotyledons of 15-day-old seedlings seeds	Regeneration	(239)
13.	*Amaranthus tricolor*	Tissue culture	MS_30_ on “hairy” roots as explant	Regeneration	(240)
14.		Tissue culture	MS_30_ + 1.5 mg/L IAA + 0.5 mg/L ZEA, MS_30_ + 1 mg/L IAA	Regeneration	(66)
15.	*Amaranthus cruentus* “Amont,” *Amaranthus hypochondriacus* “Intense Purple” and *Amaranthus* ssp. “Plenitude”	Tissue culture	$\frac{1}{2}$ strength of MS media using seeds as explant	Regeneration	(241)
16.	*Amaranthus gangeticus*	Tissue culture	MS30 + 2 mg/L NAA + 1 mg/L BA on leaves, stem and roots	Regeneration	(242)
17.	*Amaranthus tricolor* and *Amaranthus spinosus*	Tissue culture	MS media + 0.5 mg/L BAP + 0.5–1 mg/L 2,4-D on hypocotyls segments	Regeneration	(243)
18.	*Amaranthus cruentus* L. “Ficha”	Tissue culture	MS + 6-benzylaminopurine (BAP) + zeatin (ZEA), and thidiazuron (TDZ) (1, 3, and 5 mg/L) all in combination with 0.01 mg/L α-naphthaleneacetic acid (NAA), or with 1 mg/L TDZ without auxin using Epicotyl, Hypocotyls, Root and leaf segments	Regeneration	(230)
19.	*Chenopodium quinoa*	Tissue culture	2 MS + 3.0 mg/L 6-BA	For Cotyledons with petiole	(244)
			2 MS + 3.0 mg/L 6-BA and 0.1 mg/L NAA	For axillary bud	
			2 MS + 1.0 mg/L 6-BA and 0.1 mg/L NAA	Adventitious buds	
			2 MS + 0.3 mg/L 6-BA	Plantlets	
20.	*Chenopodium quinoa*	Tissue culture	MS medium + 6-benzylaminopurine (8.88 μM) and 2,4-dichlorophenoxyacetic acid (6.79 μM)	Regeneration	(245)
21.	*Chenopodium quinoa*	Tissue culture	MS medium + 0.45 μM 2,4-D using hypocotyls as explant	Regeneration	(246)
22.	*Chenopodium quinoa*	Tissue culture	MS basal medium + 0.5 mg/L 2,4-D + 0.05 mg/L BAP	Callus induction	(227)
23.	*Amaranthus* spp.	*Agrobacterium*-mediated transformation	Indirect gene transfer	Unsuccessful	(247)
24.	*Amaranthus hypochondriacus*	*Agrobacterium*-mediated transformation	*Agrobacterium tumefaciens* strains used: C58- pTiC58 and A281(pGA471)	Tissue-specific and light-inducible expression directed by a pea chlorophyll a/b-binding protein promoter in transgenic amaranth plants and their progeny	(237)
25.	*Amaranthus tricolor* L.	*Agrobacterium*-mediated transformation	Strain used: *Agrobacterium rhizogenes* A4	transgenic plants using internodes and leaf blades	(240)
26.	*Amaranthus tricolor* L.	*Agrobacterium*-mediated transformation	Strain used: *Agrobacterium rhizogenes* A4, LBA9402	Transgenic plants	(248)
27.	*Amaranthus tricolor L*.	*Agrobacterium*-mediated transformation	Strain used: *Agrobacterium tumefaciens EHA 105, LBA, 4404 (p35SGUSINT* with genes of *npt II—*kanamycin resistance and *uid*A for each strain)	Transgenic plants	(238)
28.	*Amaranthus L*.	*Agrobacterium*-mediated transformation	Strain used: *Agrobacterium tumefaciens AGL1* [*p5b5, p5d9, p5f7* with gene of hph (gene codes hygromycin-B-phosphotransferase protein)]	Transgenic plants	(249)
29.	*Amaranthus trisis* Willd. *(trisis is the* synonym of *Amaranthus dubius* Mart. ex Thell.	*Agrobacterium*-mediated transformation	Strain used: *Agrobacterium tumefacies strain EHA 105 harbouringpCAMBIA, 1301*	Transgenic plants	(250)
30.	*Amaranthus retroflexus* L.	*Agrobacterium*-mediated transformation	Strain used: *Agrobacterium tumefaciens* strain AGL0, which contained gene construction in the vector pCAMBIA, 1301 with ARGOS-like gene from *Arabidopsis thaliana* (ARL)	Transgenic plants	(251)
31.	*Amaranthus cruentus* L.	*Agrobacterium*-mediated transformation	Strain used: *Agrobacterium tumefaciens* strain AGL0, which contained gene construction in the vector pCAMBIA, 1301 with ARGOS-like gene from *Arabidopsis thaliana* (ARL)	Transgenic plants	(252)
32.	*Amaranthus caudatus* L. cv. Karmin, cv. Helios	*Agrobacterium*-mediated transformation	Strain used: *Agrobacterium tumefaciens* strain GV3101 [with *uid*A and *bar* (phosphinothricin N-acetyltransferase) genes]	Transgenic plants	(253)
33.	*Amaranthus caudatus* L.	*Agrobacterium*-mediated transformation	Strain used: *Agrobacterium rhizogenes A4*	Transgenic plants	(254)
34.	*Amaranthus caudatus*	*Agrobacterium*-mediated transformation	*Agrobacterium rhizogenes* A4 strain and *Agrobacterium tumefaciens* GV3101 strain used	Resistant plants to herbicide	(255)
35.	*Amaranthus hypochondriacus* and *Amaranthus hybridus*	*Agrobacterium*-mediated transformation	*Agrobacterium rhizogenes* were used: R1000, K599 and BVG strain	Protocol for plant regeneration via somatic embryo germination from grain amaranth transgenic hairy roots	(256)
36.	*Amaranthus cruentus* L	*Agrobacterium*-mediated transformation	Epicotyl segments by the ARGOSLIKE transgene of *Arabidopsis thaliana* controlled by the 35S promoter in the binary vector pCambia, 1301 with a selective hygromycin B resistance gene	Transgenic plants	(257)
37.	*Chenopodium quinoa*	*Agrobacterium*-mediated transformation	Two transformation systems using hairy root and leaf agroinfiltration	DNA manipulation	(225)
38.	*Chenopodium quinoa*	*Agrobacterium*-mediated transformation	Transcriptome analysis (Agrobacterium-mediated transformation protocol)	QTL mapping	(226)
39.	*Chenopodium quinoa*	*Agrobacterium*-mediated transformation	Gene-editing systems	Transgenic plants	(258)
40.	*Amaranthus hypochondriacus*	Molecular markers	SNP markers	Genetic diversity and fingerprinting	(22)
41.	*Chenopodium quinoa*	Molecular markers		Floral expressed sequenced tag (EST)	(209)
42.	*Chenopodium quinoa*	SNP assays		Fluidigm dynamic array based on KASPar genotyping	(205)
43.	*Chenopodium quinoa*	Molecular markers		Genetic diversity and fingerprinting	(210)
44.	*Amaranthus* spp.	Molecular markers	RAPD primers	Genetic diversity and fingerprinting	(24)
45.	*Chenopodium quinoa*	Molecular markers	RAPD markers	Genetic diversity and fingerprinting	(200)
46.	*Chenopodium quinoa*	Molecular markers	RAPD and ISSR markers	Genetic diversity and fingerprinting	(203)
47.	*Chenopodium quinoa*	Molecular markers Molecular markers	RAPD and ISSR markers	Genetic diversity and fingerprinting	(259)
48.	*Chenopodium quinoa*	Molecular markers	ISSR markers	Molecularly characterization of quinuamaterials	(216)
49.	*Chenopodium quinoa*	Molecular markers	ISSR markers	Genetic diversity and fingerprinting	(219)
50.	*Chenopodium quinoa*	Molecular markers	ISSR markers, SCoT DNA chloroplast markers	DNA fingerprinting and barcoding	(220)
51.	*Amaranth* spp.	Molecular markers	SSR markers	Genetic diversity	(142)
52.	*Chenopodium quinoa*	Molecular markers		Detect polymorphism in quinoa	(204)
53.	*Chenopodium quinoa, Chenopodium giganteum, Chenopodium album*	Molecular markers		Fluorescence-tagged markers analysis	(206)
54.	*Chenopodium quinoa* Willd.	Molecular markers		Genetic mapping	(203)
55.	*Chenopodium quinoa*	Molecular markers	EST-SSR markers	Polymorphism studies	(212)
56.	*Chenopodium quinoa*	Molecular markers	SSR markers	Genetic diversity and fingerprinting	(223)
57.	*Amaranthus hypochondriacus, Amaranthus cruentus* and *Amaranthus caudatus, Amaranthus hybridus*	Molecular markers	Chloroplast genome assembly	Genetic diversity and fingerprinting	(146)
58.	*Amaranthus cruentus*	Molecular markers	Chromosome-level genome assembly	To study role of specific genes with in phytic acid synthesis (an anti-nutrient)	(260)
59.	*Amaranthus hypochondriacus*	Molecular markers	Linkage mapping	QTL mapping	(189)
60.	*Amaranthus* spp.	Genetic Diversity	Gene mapping	DNA fingerprinting	(136)
61.	*Chenopodium hircinum* and wild quinoa ajara	Allozyme markers	Diversity study	Genetic diversity and fingerprinting	(194, 195)
62.	*Chenopodium quinoa*	Molecular markers	Microsatellite markers	DNA fingerprinting	(208)
63.	Tetraploid species of quinoa	Molecular markers	Flowering Locus T-Like genes were used as markers	Genetic diversity and fingerprinting	(211)
64.	*Chenopodium quinoa* Willd.	Molecular markers	DNA sequencing, RNA sequencing (CD-HITprogram)	Polyploidy study	(213)
65.	*Chenopodium quinoa* Willd.	Molecular markers	Chromosome-scale reference genome sequence	Genetic diversity and fingerprinting	(96)
66.	*Chenopodium quinoa* and *Chenopodium album*	Molecular markers	Complete chloroplast (cp) DNA analysis	PCR amplification with InDel specific primers	(215)
67.	*Chenopodium quinoa*	Molecular markers	InDel (insertion/deletion)	Whole-genome re-sequencing	(217)
68.	*Chenopodium quinoa*	Molecular markers	Molecular studies	Genetic diversity and fingerprinting	(218)
69.	Accessions of *Chenopodium quinoa* and of *Chenopodium album*	Molecular markers	SDS-PAGE protein profiling	Genetic diversity and fingerprinting	(221)
70.	*Chenopodium quinoa*	Molecular markers	Microsatellite markers	Genetic diversity and fingerprinting	(222)
71.	*Chenopodium quinoa*	Molecular markers	PacBio long-read sequencing	Genome assembly scaffolding	(261)
72.	*Chenopodium quinoa*	Molecular markers	Single nucleotide polymorphism (SNP)	QTLs (quantitative trait loci) Mapping	(214)

The new breeding technologies such as CRISPR/Cas [clustered regularly interspaced palindromic repeats (CRISPR)/CRISPR-associated protein]-based systems are very precise genome editing tools that allow editing of several genes or alleles simultaneously, thus proved to be a promising platform to insert desirable gene for nutrition enhancement, to lower saponin content and abiotic stress tolerance (262–270). Earlier work is done in potatoes to use endogenous promoters with CRISPR genome editing (271, 272). This technique can also be used to modify the advanced germplasm lines in Amaranth and Quinoa. In the genetic engineering of wheat, there is a need to optimize the codon of the Cas9 sequence with the CRISPR/Cas9 system and further use of a promoter from maize for expression (269). Similarly, as in wheat and maize, efficient genome editing methods can be used in Amaranth and Quinoa, and in future, there may be a need to use different promoters, terminators, or other elements from other sources of common plasmids from different crops. The molecular and molecular approaches discussed earlier and studies on the expression of different genes in this plant may be helpful to select suitable DNA fragments for generating improved Amaranth and Quinoa-optimized vectors in future research.

The latest technologies like RNA interference (RNAi) technology or Post-Transcriptional Gene Silencing (PTGS) can also be used to understand the genomes of Amaranthus and Quinoa, where reverse genetics is followed. By using this gene silencing technique, gene functional studies are carried out to understand functional genomics (273). This study can be used to understand the genomics of anti-nutritional factors present in both Amaranth and Quinoa.

Another new genome editing method is TILLING (Targeting Induced Local Lesions in Genomes), which proved to be the most effective and fast technology for identifying induced mutations in any gene (274). At present, large TILLING libraries typically contain up to 3,000 highly mutagenized individuals of quinoa (275). Advanced genetic screens enable the establishment and screening of much larger libraries derived from mutagenized populations (276). These big libraries help us to identify the genes for desirable characters to obtain good germplasm, reducing the load to identify individual mutations from such a large population. These are collections of all genes and provide instant access to numerous alternative functional alleles for every gene.

## 6. Conclusion

Amaranth and quinoa are superfoods, particularly for people with allergies to wheat or gluten. It can become part of the diet in developing countries like Africa, where people suffer from malnutrition. These crops can perform well in stress conditions with minimal use of resources. However, the anti-nutritional factors present in these crops like saponin in quinoa need to be tackled under focused breeding programs. The enormous variability in genetic resources available for these crops should be further utilized under selection for targeted traits. These crops have so far attained their full potential as major crops, but the breeding programs to enhance these crops are not up to the mark to achieve the place these crops should be given in the food industry. The cost of sophisticated technology and infrastructure is difficult to be available and so genetic advances under classical and molecular breeding methods are expected to modify this scenario by developing a nutrient-dense, high-performing crop that can fulfill upcoming food demand in a changing environment. Therefore, the region-specific varieties and agrotechnologies would be the key factors for their promotion and large-scale cultivation.

## Author contributions

A and MK: writing. GZ: editing nutritional traits. RC and AK: editing plant descriptors and discussion. SanS: editing genetic resources. SatS: conceptualization, review, and editing breeding approaches. All authors contributed to the article and approved the submitted version.
